# The Effect of Nebivolol on Office Blood Pressure of Blacks Residing in Sub-Saharan Africa (A Pilot Study)

**DOI:** 10.3389/fcvm.2020.613917

**Published:** 2021-01-11

**Authors:** Dike Ojji, Boni Maxime Ale, Lamkur Shedul, Ejiroghene Umuerri, Emmanuel Ejim, Chizindu Alikor, Charles Agunyenwa, Uche Njideofor, Helen Eze, Victor Ansa

**Affiliations:** ^1^Department of Internal Medicine, Faculty of Clinical Sciences, College of Health Sciences, University of Abuja and University of Abuja Teaching Hospital, Gwagwalada, Nigeria; ^2^Cardiovacular Research Unit, Department of Internal Medicine, University of Abuja and University of Abuja Teaching Hospital, Gwagwalada, Nigeria; ^3^Holo Healthcare Limited, Nairobi, Kenya; ^4^Department of Family Medicine, University of Abuja Teaching Hospital, Gwagwalada, Nigeria; ^5^Department of Internal Medicine, Faculty of Clinical Medicine, College of Health Sciences, Delta State University, Abraka, Nigeria; ^6^Delta State University Teaching Hospital, Oghara, Nigeria; ^7^Department of Internal Medicine, University of Nigeria and University of Nigeria Teaching Hospital, Enugu, Nigeria; ^8^Department of Internal Medicine, University of Port Harcourt and University of Port Harcourt Teaching, Port Harcourt, Nigeria; ^9^Department of Internal Medicine, University of Calabar and University of Calabar Teaching Hospital, Calabar, Nigeria

**Keywords:** nebivolol, efficacy, tolerability, hypertensive, black patients

## Abstract

**Introduction:** There is substantial clinical evidence that monotherapy with beta-blockers are less effective in reducing blood pressure among hypertensive Black patients compared to Whites. The highly selective beta-1 agents like nebivolol and bisoprolol have, however, been reported to be effective in reducing blood pressure in African Americans. However, results in African Americans cannot be extrapolated to native Africans because of genetic admixture and gene-environment interaction. There is, therefore, the need for us to generate data that are applicable to Africans residing in sub-Saharan Africa. We therefore decided to evaluate the efficacy and tolerability of highly selective beta-1 agent nebivolol in hypertensive Black patients residing in sub-Saharan Africa.

**Materials and Methods:** The nebivolol study was a multicenter, prospective, observational program among hypertensive patients with 4- and 8-week follow up which was conducted in 5 cities in Nigeria of Abuja, Calabar, Enugu, Oghara, and Port Harcourt. Dosages of nebivolol used in keeping with local prescribing information were 5 and 10 mg once daily each. The effectiveness of treatment was assessed by change from baseline in mean office systolic and diastolic blood pressures, and the proportion of patients achieving the therapeutic goal of <140/90 mmHg. Safety and tolerability of this medication were also assessed.

**Results:** We report the results of the 140 patients studied. The mean age and body mass index were 46.9 ± 7.3 years and 22.3 ± 5.8 kg/m^2^, respectively, and 57.1% were female. Nebivolol reduced SBP and DBP by 7.6 and 6.6 mmHg, respectively, in 4 weeks, and by 11.1 and 8.0 mm Hg, respectively, in 8 weeks. Blood pressure control was achieved in 54.8% of the patients in 4 weeks and increased to 60.4% in 8 weeks. There was no change in metabolic profile between randomization and at 8 weeks, and erectile dysfunction occurred in 1.3% of the study population.

**Conclusions:** Nebivolol 5 and 10 mg appear efficacious in Nigerian Africans with no negative metabolic effect and minimal side effect profile.

**Clinical Trial Registration:** www.ClinicalTrials.gov, Study Identification: NCT 03598673.

## Introduction

Although hypertension affects every racial group, complications such as heart failure, chronic kidney disease and cerebrovascular accidents are more prevalent in the Black population (1). For example, in the Abuja Heart Study, about seventy-five percent (75%) of hypertensive patients presenting *de novo* to a tertiary healthcare institution had one or more complications on presentation (2). In addition, there is substantial clinical evidence indicating that monotherapy with beta-blockers, angiotensin converting enzyme inhibitors and angiotensin receptor blockers are less effective in reducing blood pressure among hypertensive Black patients compared to Whites (3–5). The beta-blocker class is however a highly heterogeneous group in terms of its pharmacologic and hemodynamic properties (6, 7), with the low efficacy of beta-blockers as monotherapy being reported only with the more traditional agents like Atenolol and Metoprolol, while the highly selective beta-1 agents have been more recently reported to be effective in reducing blood pressure in both Blacks and Whites (8–10).

Although nebivolol, a highly selective beta-1 agent, has been shown to be effective in reducing blood pressure in Blacks (11), this was in African Americans with no study in African Blacks residing in sub-Saharan Africa. It might be argued that findings in African Americans can be extrapolated to African Blacks since they have the same ancestral origin. The differences in selection in previous generations, ethnic admixture, and differences in lifestyle suggest that such extrapolation may be inappropriate (12–14). We therefore, decided to study the effectiveness and safety of nebivolol in Black patients with stage 1 hypertension (systolic BP of 140–159 and/or diastolic BP of 90–99 mmHg) presenting to primary care clinics in tertiary health care facilities in Nigeria.

## Materials and Methods

### Study Design

The Nebivolol Hypertension study was a multicenter, prospective, observational program among hypertensive patients with 2-month follow-up carried out in the Cardiology units of five (5) public tertiary hospitals in Nigeria. It was conducted as a non-interventional study, and therefore study-specific patient visits, tests and monitoring were not imposed, and only data originating from routine clinical practice were collected. Therapy was prescribed according to clinician preference and on the prescribing pattern in Nigeria.

### Study Participants

Male or female patients aged 30–60 years of age with sitting office systolic BP of 140 mmHg or above and <160 mmHg, and diastolic BP of 90 mm Hg and above but less 100 mmHg and on no antihypertensive treatment for a minimum of six (6) months were recruited into the study. Patients with congestive heart failure, renal failure, coronary heart disease including chronic stable angina, myocardial infarction or acute coronary syndrome, stroke or transient ischemic attack, suspected secondary hypertension, concomitant illness, physical or mental impairment that could interfere with the effective conduct of the study and those who are pregnant or of child-bearing age who are not taking reliable contraception were excluded from the study.

### Outcome Measures

#### Primary Endpoint

The primary outcome measure was a change in office BP value from baseline to 2 months which was calculated as the difference between the mean office BP at randomization and at the end of follow up.

#### Secondary Endpoint

Other outcome measures include: the proportion of patients who achieved BP <140 mmHg systolic and <90 mmHg diastolic in 2 months, the proportion of patients who have adverse events, change in plasma fasting blood glucose and fasting lipid profile from baseline to 2 months and proportion of male patients who complain of erectile dysfunction at both the fourth and eighth weeks.

### Study Procedure

Patients were commenced on 5 mg of nebivolol at a starting dose of 5 mg once daily if the BP is not controlled (140/90 mmHg or above) after 4 weeks, the dose is doubled to 10 mg once daily.

### Blood Pressure Measurement

Office BP was measured after five (5) min of rest in the sitting position using semi-automated blood pressure devices (semi-automated blood pressure monitors (Omron MIT5 Connect). Three BP measurements were taken, and the last two values were used for the calculation of the mean. Patients had their fasting blood glucose and lipid profile assessed at baseline and the 8th week.

### Study Duration

Each patient was followed up for 2 months at the fourth and eighth weeks. Patients were still followed up till the 8th week even when study medication was discontinued. In the case of discontinuation of study medications, the reason(s) for discontinuation was recorded and reported. The study was completed when the last enrolled patient completed two (2) months of observation.

### Ethical Issues

The study was conducted according to the guidelines laid down by the International Conference on Harmonization for Good Clinical Practice (15). All investigators obtained ethical approval from their local institutional review boards which are affiliates of their national review boards. Eligible patients were approached regarding potential participation in the study, and the study purpose, procedures, risks, and potential benefits explained to them by the site investigators. Patients were given the opportunity to ask questions, and those who voluntarily agreed to participate in the study were asked to sign informed consent.

### Statistical Analysis

Due to the observational nature of the study, descriptive statistical methods were used and supplemented by calculation of confidence intervals wherever this aids interpretation. The calculation of *p*-values was used either as an aid to evaluating a specific difference of interest or as a “flagging” device applied to a large number of safety and tolerability—efficacy variables to highlight differences worth further attention. This was particularly useful for laboratory data which were subjected to quantitative analysis. Patients enrolled in the program with at least one follow-up visit, or a documented adverse event were considered analyzed.

Systolic and diastolic BP were modeled using linear mixed models fitted with restricted maximum-likelihood method which include adjustment for baseline BP, age (<45 or ≥45 years), gender, smoking status, body mass index (BMI) and we considered subject and time as random effects. Site level was not considered in the random effect because it was not adding significant value to the model, and in addition, some sites had very small sample which could bias our results if included in the model.

All other patients were included in the evaluation even if they had partially missing data. We investigated existence of interaction within model predictors, and in case of significant interaction between statistically significant predictors in the final model, we explored specific difference between means of blood pressure amongst the groups using pairwise test with Tukey adjustment.

Moreover, we presented the adjusted mean reductions in SBP and DBP in interesting subgroups.

Firstly, we constructed all models without accounting for missing data and secondly performed a sensitivity analysis, in which we performed several logistic regressions with binary variable (missing and not missing) and added other variables in the models as covariates in order to eliminate the “Missing Completely at Random” hypothesis. With the assumption that all missing data that were missing at random (MAR), we performed multiple-imputation analysis using chained equations. We generated 20 amputated data sets with a maximum of 20 iterations. Variables included in the imputation model were systolic BP, diastolic BP, age, gender, weight, height, BMI, smoking status, site including individuals. All analyses were performed with R Software 4.0.0 (The R Foundation for Statistical Computing platform).

### Management of the Study

The Trial Steering Committee consisted of the Principal Investigator, the site Investigators, the Statistician, and the representative of Micronova Pharmaceutical team. This committee was chaired by the Principal Investigator. The various site investigators were responsible for entering patients' data into paper case report forms which were subsequently entered into Microsoft data spreadsheet.

## Result

### Study Setting

One hundred and eighty-three (183) patients were screened, but one hundred and forty (140) met the inclusion criteria and these consist of sixty-four (64) patients from University of Abuja Teaching Hospital, thirty (30) patients from Delta State University Teaching Hospital, twelve (12) patients from University of Nigeria Teaching Hospital, seventeen (17) patients from University of Port Harcourt Teaching Hospital and another seventeen (17) patients from University of Calabar Teaching Hospital. The first patient was recruited 2nd February 2018 at the University of Abuja Teaching Hospital, and the last patient was followed up 3rd September 2019 at the University of Calabar Teaching Hospital.

### Baseline Characteristics of Patients

Table 1 shows the baseline characteristics of the patients. Patients on average were middle-aged (46.9 years ± 7.3), mean body mass index of 22.3 kg/m^2^ ± 13.6 with 38 (28.4%) overweight and 40 (29.9%) obese, with a female predominance (57.1%). Duration of hypertension was <5 years in majority of them (81.4%), and the mean systolic and diastolic blood pressures were at 148.2 mmHg ± 6.8 and 94.8 mmHg ± 5.3, respectively.

**Table 1:** Baseline characteristics of the patients.

**Characteristics**	**Value**
Age (*n* = 140)—Mean-year (yr)	46.9 ± 7.3
Gender, no. (%)—(*n* = 140)	
Male	60 (42.8)
Female	80 (57.1)
Body Mass Index (BMI)	
Mean—kg/m^2^	22.3 ± 13.6
Distribution—*n* (%)	
Normal	56 (41.8)
Overweight	38 (28.4)
Obesity	40 (29.9)
Smoking habit, no (%)	
Non-smoker	138 (98.6)
Smoker	2 (1.4)
Duration of hypertension	
<5 years, %	114 (81.4)
5–10 years, %	18 (12.9)
>10 years, %	8 (5.7)
Blood pressure—mmHg	
Systolic	148.2 ± 6.8
Diastolic	94.8 ± 5.3
Mean heart rate-bpm (*n* = 68)	79.3 ± 6
Fasting blood glucose—mmol/L (*n* = 75)	5.2 ± 1.9
Total cholesterol—mmol/L (*n* = 81)	5.0 ± 1.1
HDL cholesterol—mmol/L (*n* = 80)	1.4 ± 0.6
LDL cholesterol—mmol/L (*n* = 82)	3.1 ± 1.0
Triglyceride—mmol/L (*n* = 52)	1.3 ± 0.4

*HDL, High Density Lipoprotein; LDL, Low Density Lipoprotein; bpm, beats per minute*.

### Effect of Nebivolol on Blood Pressure Parameters and Pulse Rate

**Figure 1:**
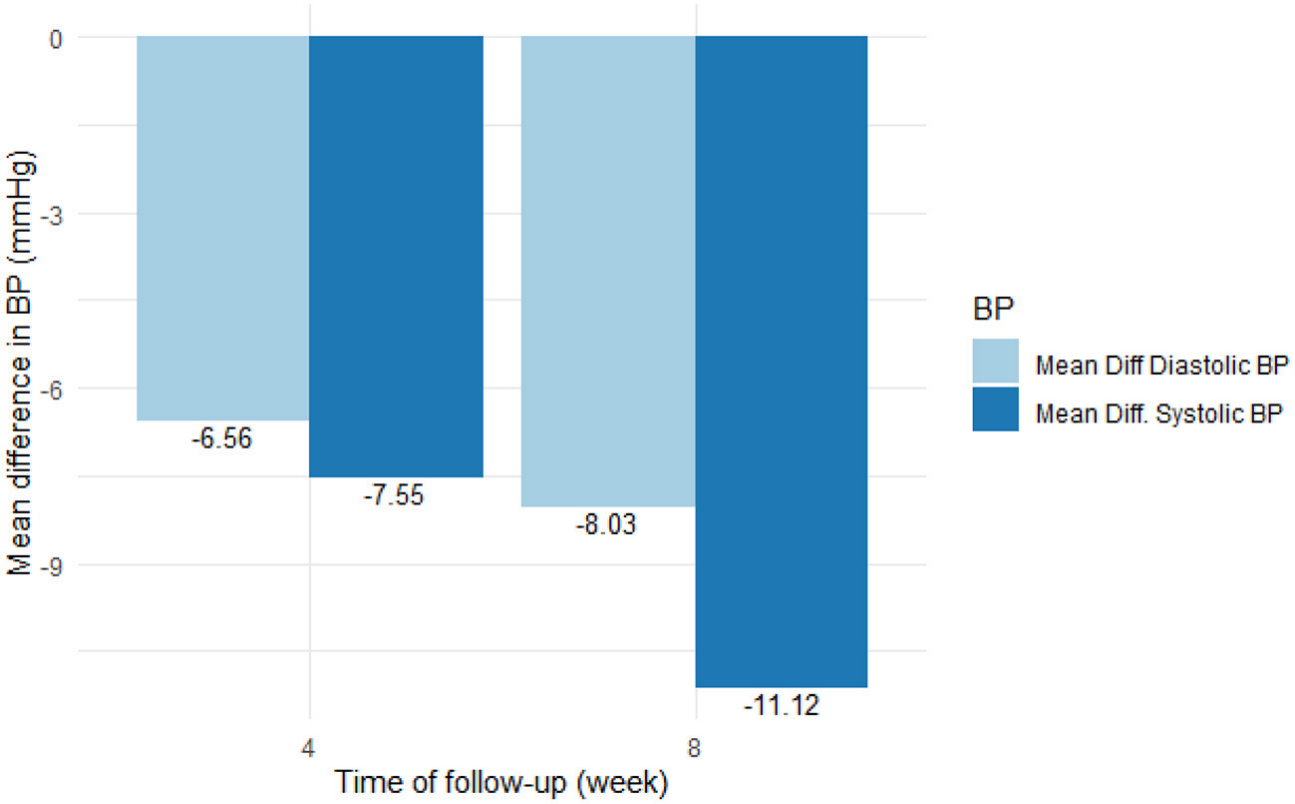
Reduction in blood pressure from baseline to 4 and 8 weeks.

Table 2 and Figure 1 show the effect of Nebivolol on blood pressure parameters and heart rate at baseline, the fourth week, and the 8 weeks. At four (4) weeks, systolic and diastolic blood pressures were significantly reduced by 7.6 mmHg (95% CI [5.0–10.1]) and 6.6 mmHg (95% CI [4.95–8.17]), respectively, and by 11.1 mmHg (95% CI [8.8–13.4]) and 8.03 mmHg (95% CI [6.45–9.62]), respectively, in the 8 weeks. BP control (BP <140/90 mmHg) was achieved in 54.8% of the patients in four (4) weeks and increased to 60.4% in eight (8) weeks.

**Table 2:** Differences in blood pressure and heart rate at baseline, 4th and 8th week.

**Variable**	**Baseline**	**4th week**	**Mean difference (95% CI)**	***P*-value**
SBP, mmHg (*N* = 140)	148.20	140.80	7.55 (5.00, 10.11)	<0.0001
DBP, mmHg (*N* = 140)	94.78	87.94	6.56 (4.95, 8.17)	<0.0001
HR, bmp (*N* = 68)	79.30	75.10	4.02 (3.32, 4.71)	<0.0001
**Variable**	**4th week**	**8th week**	**Mean difference (95% CI)**	***P*****-value**
SBP, mmHg	140.8	137.0	3.70 (1.36, 6.05)	0.002
DBP, mmHg	87.94	86.63	1.54 (−0.26, 3.35)	0.09
HR, bpm	75.10	74.40	0.77 (−0.191, 1.732)	0.1

*SBP, Systolic Blood Pressure; DBP, Diastolic Blood Pressure; HR, Heart Rate*.

Pulse rate was reduced by 4.02 beats per minute (95% CI: 3.32–4.71, *p* <0.0001) at 4 weeks and by 4.8 beats per minute (95% CI: 4.17–5.44, *p* < 0.0001) at 8 weeks. There was however no significant difference in pulse rate between the fourth and 8 weeks.

### Effect of Nebivolol on Laboratory Parameters

Table 3 shows the effect of Nebivolol on laboratory parameters. There was no change in fasting blood glucose, total cholesterol, high-density lipoprotein cholesterol, in low density lipoprotein cholesterol or triglyceride between baseline and eight (8) weeks.

**Table 3:** Comparison of mean change in blood pressure, heart rate, fasting blood glucose and fasting lipid of patients at baseline and 8th week.

**Variable**	**Baseline**	**8th week**	**Mean difference (95% CI)**	***P*-value**
SBP, mmHg	148.0	137.00	11.01 (8.80,13.44)	<0.0001
DBP, mmHg	94.8	86.60	8.03 (6.45, 9.62)	<0.0001
HR, bpm	79.30	74.40	4.8 (4.17, 5.44)	<0.0001
FBG, mmol/L	5.20	5.20	−0.125 (−0.71, 0.46)	0.70
Total cholesterol, mmol/l	5.00	4.70	0.19 (−0.15, 0.54)	0.15
LDL cholesterol, mmol/l	3.10	2.90	0.21 (−0.12 0.54)	0.21
HDL cholesterol, mmol/l	1.40	1.30	0.07 (−0.13, 0.27)	0.50
TG, mmol/l	1.30	1.20	0.04 (−0.12, 0.19)	0.60

*SBP, Systolic Blood Pressure; DBP, Diastolic Blood Pressure; FBG, Fasting Blood Glucose; LDL, Low Density Lipoprotein; HDL, High Density Lipoprotein; TG, Triglyceride*.

### Factors Influencing Blood Pressure Response to Nebivolol in Patients

**Figure 2:**
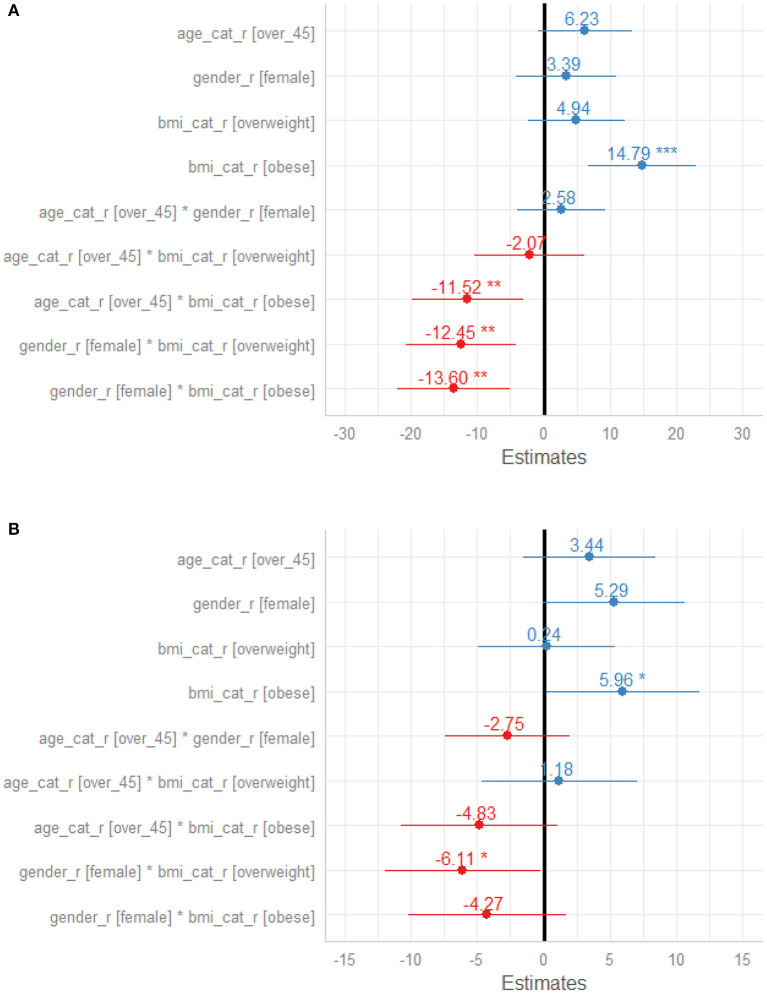
**(A)** Forest plot of factors influencing Systolic Blood Pressure response to Nebivolol treatment. **(B)** Forest plot of factors influencing Diastolic Blood Pressure response to Nebivolol treatment.

In a linear mixed model as shown in Figures 2A,B, obesity was significantly associated with changes in Systolic BP, but there was significant interaction between obesity and gender (95% CI; −20.67, −6.53, *p* = 0.001), overweight and gender (95% CI; −19.36, −5.54, *p* = 0.04). Therefore, the effect of BMI on systolic BP depend significantly on gender, but gender alone was not significantly associated with systolic BP (*p* = 0.087). A *post-hoc* test, exploring specific difference between male with normal weight, male with overweight, male with obesity and respective female subgroups revealed that the changes in SBP in male with overweight compared to female with overweight, in male with obesity compared to female with obesity and in male with obesity compared to female with overweight were significantly different. The adjusted mean reduction of SBP from baseline to 8 weeks follow-up was not statistically different in male regardless of the BMI status and these results were similar female regardless of their BMI status (Table 4). Similarly, obesity was significantly associated with changes in diastolic BP (*p* = 0.044) but there was no interaction with gender (95% CI; −9.27 0.73, *p* = 0.163) or age above 45 years (95% CI; −9.80 0.14, *p* = 0.113). The adjusted mean reduction of SBP from baseline to 8 weeks follow-up for male was 8.2 mmHg (95% CI; 7.00 9.40) and 8.1 mmHg (95% CI; 6.90 9.30) for female (Table 5).

**Table 4:** Adjusted mean reductions in Systolic Blood Pressure (SBP) in subgroups gender and BMI.

**Subgroups**	**Baseline**	**4th week**	**Adjusted mean reduction (95% CI)**
Male with normal weight	146	138	8 (6.68, 9.32)
Male with overweight	150	142	8 (6.70, 9.30)
Male with obesity	155	147	8 (6.66, 9.34)
Female with normal weight	151	142	9 (7.65, 10.35)
Female with overweight	142	134	8 (6.69, 9.31)
Female with obesity	146	138	8 (6.74, 9.26)
	**4th week**	**8th week**	**Adjusted mean reduction (95% CI**
Male with normal weight	138	135	3 (1.68, 4.32)
Male with overweight	142	139	3 (1.72, 4.28)
Male with obesity	147	144	3 (1.66, 4.34)
Female with normal weight	142	139	2 (0.64, 3.36)
Female with overweight	134	131	3 (1.69, 4.31)
Female with obesity	138	135	3 (1.73, 4.27)
	**Baseline**	**8th week**	**Adjusted mean reduction (95% CI)**
Male with normal weight	146	135	11 (9.68, 12.32)
Male with overweight	150	139	11 (9.72, 12.28)
Male with obesity	155	144	11 (9.66, 12.34)
Female with normal weight	151	139	12 (10.65, 13.35)
Female with overweight	142	131	11 (9.69, 12.31)
Female with obesity	146	135	11 (9.74, 12.26)

**Table 5:** Adjusted mean reductions in Diastolic Blood Pressure (DBP) of subgroup gender.

**Subgroups**	**Baseline**	**4th week**	**Adjusted mean reduction (95% CI)**
Male	94.5	87.7	6.8 (5.6, 8.0)
Female	94.9	88.1	6.8 (5.6, 8.0)
	**4th week**	**8th week**	**Adjusted mean reduction (95% CI)**
Male	87.7	86.3	1.4 (0.20, 2.6)
Female	88.1	86.8	1.3 (0.10, 2.5)
	**Baseline**	**8th week**	**Adjusted mean reduction (95% CI)**
Male	94.5	86.3	8.2 (7.00, 9.40)
Female	94.9	86.8	8.1 (6.90, 9.30)

A sensitivity analysis that included all patients who were treated in all study sites after multiple imputations confirmed these patterns in treatment effects on systolic BP and diastolic BP.

### Side Effect Profile

Table 6 shows the side effect profile of Nebivolol. Side effects occurred in 6 (4.4%) of the patients with 3 (2.2%) discontinuing their medication. The commonest side effect was dizziness in 5 (3.3%), followed by headaches in 3 (2.2%). Erectile dysfunction occurred in 2 (1.2%) of the study population. Four (2.7%) of the patients had more than one side effect occurring in them.

**Table 6:** Adverse events.

**Adverse events**	**Percentage (%)**
Dizziness	5 (3.3)
Headaches	3 (2.2)
Dry cough	3 (1.8)
Difficulty in breathing	3 (1.8)
Erectile dysfunction	2 (1.2)

*3 (2.2%) stopped their medications because of side effects*.

## Discussion

This study has demonstrated that nebivolol which is a cardio-selective beta-1 blocker with vasodilating effects given once-daily as 5 or 10 mg effectively reduced both systolic and diastolic blood pressure in Blacks with stage I hypertension who are residing in sub-Saharan Africa. In addition, 5 or 10 mg nebivolol resulted in response rates of >55%. These findings are of clinical importance, especially because of the excessive burden of hypertension and its complications and the low rates of blood pressure control in sub-Saharan Africa (2, 16). The results imply that beta-1-selective beta-blockers like nebivolol apart from being used as monotherapy in grade 1 hypertension could be the fourth class of medication to be considered after maximum doses of a combination of long-acting calcium channel blockers, thiazide diuretics and renin-angiotensin-aldosterone blockers have been used in this population group. Current hypertension guidelines prefer aldosterone antagonist or alpha-1 blocker as the fourth class of medication (17, 18).

The findings in this study are also very important as Physicians both in sub-Saharan Africa, Europe and the Americas are less likely to prescribe beta-blockers as monotherapy to Blacks compared to white patients in the general community (19, 20). This prescription pattern is influenced by data suggesting that beta-blockers as a class are less effective than other agents in African Americans and that they may be associated with poor tolerability and adverse metabolic effects (21, 22). The present study provides evidence to suggest that this generalization may not apply to beta-1 selective agents like nebivolol that has demonstrated significant antihypertensive efficacy and satisfactory tolerability in this patient population.

At 8 weeks, nebivolol reduced systolic blood pressure by 11.1 mmHg and diastolic blood pressure by 8.03 mmHg from baseline similar to values recorded in earlier studies in African Americans (8–11). Also similar to previous study in African Americans, nebivolol reduced pulse rate significantly at the fourth and eighth weeks compared to baseline (11). It has been suggested that the important pharmacologic attribute that may contribute to nebivolol's effectiveness in black patients as demonstrated in this and earlier studies is the vasodilating properties, contrary to the mode of action of cardio-selective non-vasodilating beta-blockers, like atenolol which primarily reduce BP by reducing cardiac output (6, 23). This type of mechanism of action by non-vasodilating beta-blockers does not favor hypertensive Black patients, who often have a decreased cardiac output with increased peripheral vascular resistance (24–26). These hemodynamic actions by nebivolol are also shared by other vasodilatory beta-blockers (27, 28). The efficacy of nebivolol at both the fourth and eighth week in reducing blood pressure can also be attributed to the young population studied. Clinical studies have shown that young African American patients respond better to beta-blocker therapy than the elderly patients as a result of their tendency toward normal renin levels with renin levels eventually declining with age (29, 30). In this study, plasma renin levels were not measured.

Apart from being at higher risk for cardiovascular and renal disease, it has been shown that black patients also generally report poorer antihypertensive medication adherence compared with the general hypertensive population (31, 32), and such poor compliance with therapy is often associated with poor tolerability (33). The good tolerability profile of nebivolol in this study could have accounted for the high compliance rate in this study, with only 2.4% of the patients discontinuing their medications because of side effects.

Unlike traditional beta-blockers, nebivolol had no negative metabolic impact on glucose and lipid profile as demonstrated in this study. LDL cholesterol was however found to be significantly lower at 8 weeks in our study. The mechanism of this reduction is not clear and has to be explored further.

One important strength of this trial is that the results were adjusted to account for the heterogeneity of the study population and potential confounding variables such as age, sex, diabetes, and obesity, which are known to determine BP response to treatment within racial groups (33).

The main limitations of this study include the apparently small sample size and the low proportion of diabetic patients who were enrolled in the study, making the extrapolation of results to this population rather difficult. In addition, plasma renin activity and adrenaline levels were not measured making it difficult to fully define the mechanism of action of nebivolol in this population.

In conclusion, nebivolol monotherapy is safe and effective in lowering BP in hypertensive black patients residing in sub-Saharan Africa. Nebivolol also has favorable metabolic effect in this population. Studies in a larger population is however needed to confirm these findings.

## Data Availability Statement

The raw data supporting the conclusions of this article will be made available by the authors, without undue reservation.

## Ethics Statement

The studies involving human participants were reviewed and approved by University of Abuja Teaching Hospital, Human Ethics Committee. The patients/participants provided their written informed consent to participate in this study.

## Author Contributions

DO was involved in the design and the management of the study and drafted the initial manuscript. BA was involved in the analyzing the data and critical review of the manuscript. LS, EU, EE, CAl, HE, CAg, UN, and VA were involved in data collection and critical review of the manuscript. All authors contributed to the article and approved the submitted version.

## Conflict of Interest

BA was employed by the company Holo Healthcare limited, Nairobi. The remaining authors declare that the research was conducted in the absence of any commercial or financial relationships that could be construed as a potential conflict of interest.
